# Intermittent Diazepam versus Continuous Phenobarbital to Prevent Recurrence of Febrile Seizures: A Randomized Controlled Trial

**Published:** 2016

**Authors:** Mohammadreza SALEHIOMRAN, Seyed Mohammad HOSEINI, Ali GHABELI JUIBARY

**Affiliations:** 1 Pediatric Neurologist, NonCommunicable Pediatric Diseases Research Center, Babol University of Medical Sciences, Babol, Iran; 2 Pediatrician, Non-Communicable Pediatric Diseases Research Center, Babol University of Medical Sciences, Babol, Iran; 3 Department of Neurology, Mashhad University of Medical Sciences, Mashhad, Iran

**Keywords:** Prophylaxis, Febrile Seizures, Diazepam, Phenobarbital

## Abstract

**Objective:**

Febrile seizure is the most common neurologic problem in children between 3 months to 5 years old. Two to five percent of children aged less than five yr old will experience it at least one time. This type of seizure is age dependent and its recurrence rate is about 33% overalls and 50% in children less than one yr old. The prophylactic treatment is still controversial, so we conducted a randomized controlled clinical trial to find out the effectiveness of continuous phenobarbital versus intermittent diazepam for febrile seizure.

**Materials & Methods:**

This clinical trial was conducted in the Department of Pediatric Neurology, Babol University of Medical Sciences, Babol, Iran between March 2008 and October 2010. All children from 6 month to 5 yr old referred to Amirkola Children’s Hospital, Babol, Iran were enrolled in the study. Children with febrile seizure that had indication for prophylaxis but did not receive any prophylaxis previously were enrolled in the study. For prophylactic anti convulsion therapy, patients were divided randomly in two groups. One group received continuous phenobarbital and another treated with intermittent diazepam whenever the children experienced an episode of febrile illness for up to one year after their last convulsion.

**Results:**

Of all 145 studied cases, the recurrent rate in children under prophylaxis with diazepam was 11/71 and in phenobarbital group was 17/74. There was no significant difference in the recurrence rate in both groups.

**Conclusion:**

There was no significant difference in the effectiveness of phenobarbital and diazepam in prevention of recurrent in febrile seizure and we think that in respect of lower complication rate in diazepam administration, it cloud be better choice than phenobarbital.

## Introduction

Febrile Seizure (FC) is one of the most common transient neurologic disorders in infants and childhood. Its prevalence is about 2-5% and the mean age at onset is 3 month to 5 yr old (1). Even the prognosis is good for the most patients, but seizures are upsetting to the children and parents. Prevention of the next seizures is important for prevention of brain hypoxic damage and sudden fall related injuries (2, 3). Fortunately, in most cases, there is no need to treatment but in some cases with high risk for recurrence prophylaxis must be considered. The overall rate of recurrence after the first episode is about 30%-37% but in child less than 1 year old, it could be about 50% (4). Some recurrence risk factors include age less than one yr old, the history of febrile seizure in fist – degree relatives of seizures occur at temperatures less than 39°C and when there is low Interval between fever and seizure (5). In child with one episode of recurrence, the risk of subsequent recurrence increases and 9-17% of them will experience more than 3 times recurrence. Three quarters of it occurs within the first year after seizure (5). Intermittent prophylaxis with oral diazepam during the first 3 days of febrile illnesses is associated with less complication such as lethargy and ataxia (6) but permanent prophylaxis with oral phenobarbital may be associated with complications like behavioral disturbances, irritability, hyperactivity and decreased of cognitive function (7). Use of rectal diazepam during an episode of seizure is another method of prophylaxis not often considered because of its failure to prevent the onset of seizure and its age related limitation. It is used to prevent seizure in 3 to 5 yr-old children and thus prevent prolongation of seizures and its recurrence until next 12 hours (6, 8). To evaluate the efficacy of different anticonvulsive regimes for preventing febrile seizures, many randomized clinical trials muse be done. In this study, we conducted a single blind, randomized clinical trial to compare effectiveness of intermittent oral diazepam versus continuous phenobarbital in children with febrile convolution.

## Materials & Methods

This single blind randomized clinical trial, registered as RCT of code No. CT2015080223393N2, was done in the Department of Pediatric Neurology, Babol University of Medical Sciences, Babol, Iran between March 2008 and October 2010. All children 6 months to 5 yr old with recurrent simple FC(≥ 3 episode) or complex FC that did not receive any anticonvulsive drug ,were enrolled into the study. Simple FC were defined as brief (< 15 minutes), generalized seizure in association with fever and only once during a 24-hour period. Childe with history of neonatal seizure, seizure without fever, chronic disease related electrolytes imbalance and history of anticonvulsive consumption, were excluded. The study was approved by the regional Ethics Committee of Babol University of Medical Sciences. After getting informed consent of child parents, all of them randomly were divided in tow prophylaxis groups. One group received oral phenobarbital (Tab 15 and 60 mg), 3-5 mg/kg/day in tow divided doses for at least one year and another group was recommended to use oral diazepam 0.33 mg/kg/TDS during febrile illness for two days without antipyretics consumption. We recommended that the child should be admitted to hospital if new convulsions occurred. Basic data on the mother’s pregnancy, the child’s birth, neonatal period, sex, age, previous febrile convulsions with detailed description of first convulsion, and epilepsy in parents or siblings were obtained from medical document and parents. All cases were fallowed regularly up to one year after their convulsion. In each monthly call, parents were questioned about occurrence of fever, new febrile convulsions, compliance with instructions, drug related side effects, and assesses the child’s clinical progress, including possible side effects of medication. In case of unable to follow the patient, or other diagnoses achieved, he/she was excluded. The main outcome was report of febrile seizures by the parents. Data were analyzed using the chi-squared test or Fisher’s exact test using the SPSS version 21 (Chicago, IL, USA). P less than 0.05 were considered significant.

## Results

**Fig 1:**
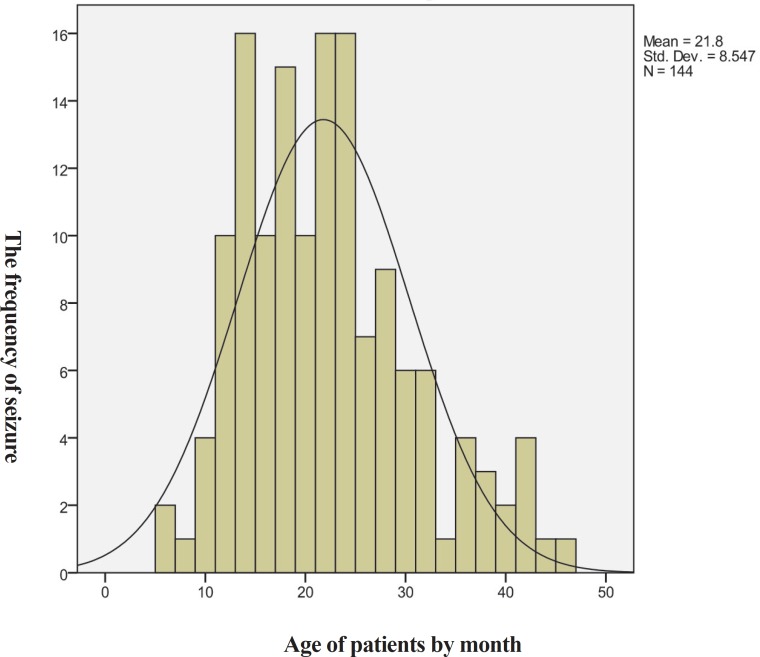
The age of patient follows normal distribution curve

Of all 154 studied patients, nine patients excluded because of the aforementioned causes. Out of the remaining 145 patients, 71 were in the diazepam and 74 in the phenobarbital group. The mean age of them was 22.61±9.11 and 20.59±7.93 months respectively (P =0.158). There was no significant difference between the mean age of cases in both groups and according to the Fig 1, the age of patient followed normal distribution curve. Table 1 shows the demographic data of patient in both groups and according to Fishers Exact Test result, there was no significant difference between the base line variable in both groups. In one year’s fallow up of patients, we had recurrence in 11 (15.5%) cases of diazepam group and in 17 cases (23%) of phenobarbital group. There was no significant difference between the recurrence rate in both groups (P=0.296). Side effects of phenobarbital like hyperkinesia, irritability, and restlessness were observed in some patients but diazepam related side effects except sedation were not seen.

**Table1:** Clinical Data Phenobarbital versus Diazepam

**Group**	**Phenobarbital n (%)**	**Diazepam n (%)**	**Fishers Exact Test**
**Male**	46 (62.2)	41(57.7)	0.614
**Female**	28 (37.8)	30(42.3)	
**Recurrent Febrile Seizure**	50 (67.6)	50(70.4)	0.724
**Complex Febrile Seizure**	24 (32.4)	21(29.6)	

## Discussion

We did not find any significant difference of two studied prophylaxis protocols but the recurrence rate by diazepam was less than phenobarbital (15.5% vs. 23%). Most previous studies have compared two drugs with placebo and clinical trials comparing these together are fewer. In Italy, the rate of febrile seizure recurrence with oral diazepam was about 11.1% compared to the 30.7% recurrence rate in those who did not receive any treatment, was substantially reduced. Lack of timely detection of fever by parents was one of the diazepam administration problems in addition to ataxia and lethargy as side effects (9). Rose and colleagues (11) conducted a study on clobazam and the ataxia rate was less than diazepam. In a study, recurrent febrile seizure risk, significantly reduced with intermittent oral diazepam versus placebo or phenobarbital versus placebo (10). However, parents and families should be supported with adequate contact details of medical services and information on recurrence, first aid management and, most importantly, the benign nature of the phenomenon (10). Efficacy of diazepam in preventing febrile seizure compared with placebo is obvious, but it was not statistically significant (2, 3).

**In conclusion**, Diazepam is a safe and quickly absorbed anticonvulsant that its peak serum level in oral consumption, reach more rapidly in children and we think in respect of the well-known side effects of phenobarbital, it can be a good choice for prophylaxis of febrile seizure.

## References

[1] Nelson KB, Ellenberg JH (1978). Prognosis in children with febrile seizures. Pediatrics.

[2] Rosman NP, Colton T, Labazzo J, Gilbert PL, Gardella NB, Kaye EM (1993). A controlled trial of diazepam administered during febrile illnesses to prevent recurrence of febrile seizures. N Engl J Med.

[3] Autret E, Ployet JL (2002). Traitement des convulsions fébriles. Arch Pédiatr.

[4] Annegers JF, Hauser WA, Shirts SB (1987). Factors prognostic of unprovoked seizure after febrile convulsion. N Engl J Med.

[5] Sulzbacher S, Farwell JR, Temkin N, Lu AS, Hirtz DG (1999). Late cognitive effects of early treatment with phenobarbital. Clin Pediatr.

[6] Millichap JG, Colliver JA (1991). Management of febrile seizures: survey of current practice and phenobarbital usage. Pediatr Neurol.

[7] Thilothammal N, Krishnamurthy PV, Kamala KG, Ahamed S, Banu K (1993). Role of phenobarbitone in preventing recurrence of febrile convulsions. Indian Pediatr.

[8] Farwell JR, Lee YJ, Hirtz DG, Sulzbacher SI, Ellenberg JH, Nelson KB (1990). Phenobarbital for fibril seizures: effects on intelligence and on seizure recurrence. N Engl J Med.

[9] Verroti A, Latini G, Trotta D (2004). Intermittent oral DZP prophylaxis in FC: its effectiveness for FC. Eur J Ped Neurol.

[10] Offringa M, Newton R (2012). Prophylactic drug management for febrile seizures in children. Cochrane Database Syst Rev.

[11] Rose W1, Kirubakaran C, Scott JX (2005 ). Intermittent Clobazam therapy in FC. Indian J Pediatr.

